# Weight loss, changes in body composition and inflammatory status after a very low-energy ketogenic therapy (VLEKT): does gender matter?

**DOI:** 10.1186/s12967-024-05733-3

**Published:** 2024-10-19

**Authors:** Giovanna Muscogiuri, Ludovica Verde, Evelyn Frias-Toral, Claudia Reytor-González, Giuseppe Annunziata, Mattia Proganò, Silvia Savastano, Daniel Simancas-Racines, Annamaria Colao, Luigi Barrea

**Affiliations:** 1 Diabetologia e Andrologia, Dipartimento di Medicina Clinica e Chirurgia, Unità di Endocrinologia, Università Degli Studi di Napoli Federico II, Via Sergio Pansini 5, 80131 Naples, Italy; 2 Centro Italiano per la cura e il Benessere del Paziente con Obesità (C.I.B.O), Diabetologia e Andrologia, Dipartimento di Medicina Clinica e Chirurgia, Unità di Endocrinologia, Università Degli Studi di Napoli Federico II, Via Sergio Pansini 5, 80131 Naples, Italy; 3 Cattedra Unesco “Educazione Alla Salute e Allo Sviluppo Sostenibile”, University Federico II, Naples, Italy; 4 Department of Public Health, University of Naples Federico II, Via Sergio Pansini 5, 80131 Naples, Italy; 5 School of Medicine, Universidad Católica de Santiago de Guayaquil, Av. Pdte. Carlos Julio Arosemena Tola, Guayaquil, 090615 Ecuador; 6 Facultad de Ciencias de la Salud Eugenio Espejo, Centro de Investigación en Salud Pública y Epidemiología Clínica (CISPEC), Universidad UTE, Quito, 170129 Ecuador; 7 Facoltà Di Scienze Umane, Della Formazione E Dello Sport, Università Telematica Pegaso, Via Porzio, Centro Direzionale, Isola, F2, 80143 Naples, Italy; 8 Dipartimento di Benessere, Nutrizione e Sport, Centro Direzionale, Università Telematica Pegaso, Via Porzio, Isola, F2, 80143 Naples, Italy

**Keywords:** Sex, Gender, Obesity, Weight loss, Very low-calorie ketogenic diet, VLCKD, Very Low-Energy Ketogenic Therapy (VLEKT), Ketogenic diet, Nutrition, Body composition

## Abstract

**Background:**

Considering differences in body composition and inflammatory status between sexes, as well as recent recommendations advocating for personalized dietary approaches, this study aimed to explore how sex influences weight loss, changes in body composition, and inflammatory status in subjects with grade I and II obesity undergoing a 45-day of the Very Low-Energy Ketogenic Therapy (VLEKT).

**Methods:**

Participants (21 premenopausal females and 21 males), included in the study adhered to the 45-day of the VLEKT and underwent assessments of anthropometric parameters (weight, height, body mass index—BMI –, and waist circumference), body composition via bioelectrical impedance analysis, and inflammatory status measured by high sensitivity C-reactive protein (hs-CRP) levels at baseline and post-intervention.

**Results:**

At baseline, premenopausal females and males did not differ in BMI (*p* = 0.100) and hs-CRP levels (*p* = 0.948). Males demonstrated overall larger benefits than premenopausal females from the VLEKT in terms of weight loss (Δ% = − 11.63 ± 1.76 *vs *− 8.95 ± 1.65 kg, *p* < 0.001), fat mass (Δ% = − 30.84 ± 12.00 *vs* -21.36 ± 4.65 kg, *p* = 0.002), and hs-CRP levels (Δ% = − 41.42 ± 21.35 *vs *− 22.38 ± 17.30 mg/L, *p* = 0.003). Of interest, in males phase angle values are statistically improved compared to female (Δ% = 17.11 ± 9.00 *vs* 7.05 ± 3.30°, *p* < 0.001).

**Conclusion:**

These findings underscore the importance of considering sex-specific responses in personalized obesity treatment strategies, particularly dietary interventions like VLEKTs.

**Graphical Abstract:**

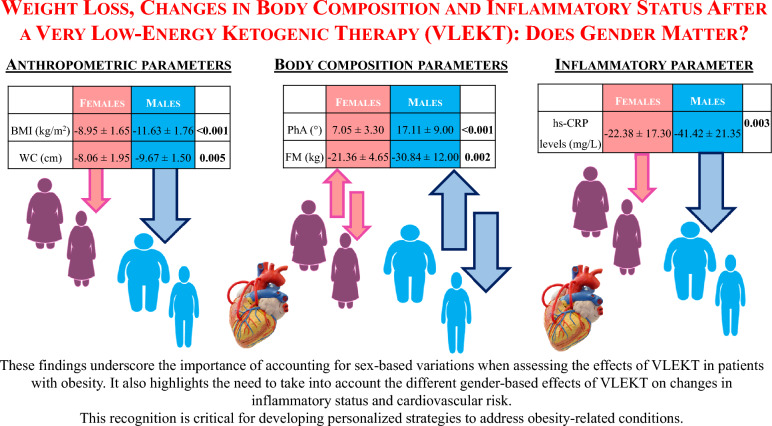

**Supplementary Information:**

The online version contains supplementary material available at 10.1186/s12967-024-05733-3.

## Background

Obesity is a multifaceted condition, yet considerations for sex differences are often overlooked in its prevention and clinical management [1]. Sex, a classification rooted in physiological and biological attributes arising from chromosomal complement [1], holds increasing recognition for its impact on health [2]. Integrating sex-specific considerations into clinical research is imperative for providing comprehensive and accurate information and care. Notably, sex disparities in obesity manifest across various aspects, encompassing prevalence, comorbidities, and treatment responses [3, 4]. Recognizing sex differences as a modifier of weight loss in individuals with obesity is pivotal for tailoring personalized treatment approaches.

Globally, obesity affects over 650 million individuals, contributing to a rising burden of metabolic, cardiovascular, and neoplastic conditions [5]. Prevalence rates indicate that 15% of females and 11% of males are affected by obesity [5], with females exhibiting a higher inclination to seek treatment, potentially influenced by socio-cultural factors [6, 7]. Consequently, males are frequently underrepresented in clinical studies.

Sexual dimorphism influences several features of obesity, including body composition, endocrine hormonal profiles, and low-grade chronic inflammation [7, 8]. Women tend to accumulate more subcutaneous fat in the iliofemoral regions, while men show a greater tendency for visceral fat accumulation in the abdomen. In addition, women may have estrogen-influenced hormone levels, which can alter fat accumulation and distribution, while men may have higher testosterone levels associated with greater muscle mass and lower body fat percentage [7, 8]. Finally, large epidemiological studies have consistently reported a clear sex dimorphism in C-reactive protein levels, with women exhibit higher levels of inflammation associated with obesity [9–11].

Studies exploring whether gender differences impact weight loss following medical and surgical treatments have yielded conflicting results [12, 13]. Very low-carbohydrate ketogenic diets (VLCKDs) have emerged as a dietary intervention for obesity management, demonstrating efficacy with or without metabolic comorbidities [14–17]. VLCKDs facilitate significant, well-tolerated, and rapid weight loss while preserving lean mass, enhancing muscle strength, and reducing fat mass [18]. Characterized by minimal carbohydrate and calorie intake (< 50 g/day and 800 kcal/day, respectively), VLCKDs induce a metabolic shift towards ketone body production by reducing insulin levels and increasing glucagon [19].

Recent consensus from the Italian Society of Endocrinology (SIE) underscores the utility of ketogenic nutritional therapy (KeNuT) incorporating meal replacements for managing obesity and associated metabolic disorders [20]. Nutritional ketosis induced by VLCKDs has been associated with accelerated weight loss compared to low-calorie non-ketogenic diets, without subsequent weight regain [20]. Consequently, VLCKDs have proven effective in achieving pre-operative weight loss in individuals with severe obesity preparing for bariatric surgery, thereby mitigating perioperative risks [21]. Of interest, very recently in order to avoid confusion with very low carbohydrate diets, a new definition and more appropriate acronym has been proposed by the panel of experts "KetoNut" of the Italian Society of Nutraceutics (SINut) and the Italian Association of Dietetics and Clinical Nutrition (ADI), which is more appropriate and avoids confusion with very low carbohydrate diets: Very Low Energy Ketogenic Therapy (VLEKT) [22].

Interestingly, the studies that have investigated the effects of VLEKT in various clinical settings, although stratifying by gender, have evaluated the results as a whole, on the total population, thus generalising the effectiveness of the nutritional intervention to a particular class of subjects or, at least, have not focused on any differences between males and females. To our knowledge, only two studies have focused on the gender-specific effect of VLEKT with meal replacement, with a focus on liver function and weight [23, 24]. Of these, in particular, one study showed that following VLEKT in males there was a greater reduction in weight and improvement in liver parameters than in females; however, the dietary intervention was not formulated exclusively with meal replacement according to the KeNuT protocol, but rather alternating the intake of protein powder preparations and fresh food, while maintaining the nutritional characteristics of VLEKT [23]. In the other study, instead, VLEKT was formulated with the use of meal replacement but, although gender differences in changes in liver parameters were reported after 8 weeks of dietary intervention, no attention was paid to the different weight loss between males and females [24]. Overall, therefore, this literature suggests a lack of specific evidence on the different effects of VLEKT formulated according to the KeNuT protocol on weight, body composition and inflammation according to gender.

Considering gender differences in body composition, inflammatory status, and recent guidelines advocating for personalized dietary therapies utilizing meal replacements in VLEKTs by the SIE, this study aimed to investigate the impact of gender on weight loss, body composition changes assessed via bioelectrical impedance analysis (BIA), and inflammatory status evaluated through high sensitivity C reactive protein (hs-CRP) levels in adults with grade I and II obesity without metabolic comorbidities undergoing VLEKTs with meal replacements over a 45-day period.

## Materials and methods

### Study population

Males and premenopausal females, 21 – 48 years of age, with obesity (30.07 – 39.79 kg/m^2^) and without comorbidities, were recruited from the Unit of Endocrinology, Obesity Unit (*Centro Italiano per la Cura e il Benessere del Paziente con Obesità, “C.I.B.O.”* and European Association for the Study of Obesity, Collaborating Centre for Obesity Management, “EASO-COMs”), Clinical Medicine and Surgery Department, University of Naples Federico II; Naples, Italy.

We decided to compare males with premenopausal females to maximize differences due to sex hormones. All participants had a history of failed dietary attempts and the desire to lose weight. Males and females included in the study were matched on a group basis for age and BMI. We excluded participants who had endocrine disorders, severe obesity (BMI ≥ 40.0 kg/m^2^), cardiovascular events or type 1 or type 2 diabetes, smokers, those who practiced regular daily physical activity (at least 30 min a day), pregnant women, and those who used medications that could affect the dependent variables under study (such as anti-inflammatory drugs) within the past month. Furthermore, all patients with absolute contraindications to the prescription of VLEKT were excluded, Table 1.

**Table 1:** Contraindications to VLEKT of SIE

Pregnancy and breastfeeding kidney failure	
48 h prior to elective surgery or invasive procedures and perioperative period Moderate-to-severe chronic kidney disease	
Liver failure	
Rare disorders: carnitine palmitoyltransferase deficiency, carnitine deficiency, porphyria, carnitine-acylcarnitine translocase deficiency, pyruvate carboxylase deficiency and mitochondrial fatty acid β-oxidation disorders	
Respiratory failure unstable angina, hearth failure (NYHA III–IV), recent stroke or myocardial infarction (< 12 months), and cardiac arrhythmias	
Eating disorders and other severe mental illnesses, alcohol, and substance abuse	
Type 1 diabetes mellitus, latent autoimmune diabetes in adults, β-cell failure in T2DM, use of SGLT2 inhibitors	
Active/severe infections and frail elderly patients	

SIE, Italian Society of Endocrinology; NYHA, New York Heart Association; T2DM, type 2 diabetes mellitus; SGLT, sodium-dependent glucose cotransporters

**Fig. 1:**
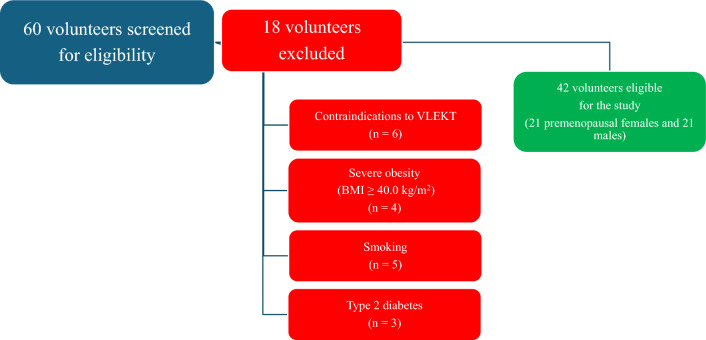
Flow-chart of study participants

A total of 42 of the 60 volunteers seen at screening met the inclusion criteria. Figure 1 shows the flowchart of the study participants. The guidelines of the Declaration of Helsinki were used to conduct the present study. All procedures involving human subjects were approved by the Federico II Ethical Committee on Human Experimentation. All patients were informed of the study's design and purpose, subsequently giving their informed consent.

### Study protocol

At baseline, all participants underwent endocrinological and nutritional visits to evaluate exclusion criteria in research. When enrolled, the endocrinologist, following EASO's guidelines [19], collected a complete clinical history and ruled out contraindications to prescribing VLEKT. Afterwards, both groups received nutritional counselling that included assessments of anthropometric parameters, assessments of body composition, and the VLEKT protocol to be followed for 45 days. The follow-up visit was agreed upon after 45 days, during which all subjects underwent endocrinological and nutritional examinations. Weekly, an experienced nutritionist telephoned both women and men to check adherence to nutritional protocols, assessing and recording values of β-hydroxybutyrate from capillary blood using test strips (Optium Xceed Blood Glucose and Ketone Monitoring System; Abbott Laboratories, Chicago, IL, USA). State of ketosis was verified as present/absent (yes/no). In addition, the nutritionist recorded any changes in physical activity levels and/or food and beverage intake that were outside the VLEKT protocol. Physical activity levels were assessed by participants' self-reported adherence to the exercise regimen (yes/no response). Physical activity noncompliance (no) was defined as failure to meet the minimum requirement of at least 30 min of aerobic exercise per day, as previously reported in other studies [25, 26]. Participants were asked not to start any kind of physical activity throughout the duration of the study.

### Anthropometric measurements

Anthropometric measurements were taken, after an overnight fast, at a time between 8 a.m. and 10 a.m., by the same trained health worker. During the measurements, participants wore light clothing and no shoes. Body weight was determined with a scale (Seca 711; Seca, Hamburg, Germany) to the nearest 0.1 kg, while height was measured with a wall stadiometer (Seca 711; Seca, Hamburg, Germany) to the nearest 0.5 cm. BMI was calculated as weight divided by the square of height [weight (kg)/height^2^ (m^2^)]. Based on BMI values, females and males were classified as overweight (25.0–29.9 kg/m^2^), grade I obesity (30.0 – 34.9 kg/m^2^), grade II obesity (35.0 – 39.9 kg /m^2^), and degree III obesity (≥ 40.0 kg/m^2^) [27]. However, only participants with a BMI between 30.0 and 39.9 kg/m^2^ participated in the study. Waist circumference (WC) was measured in subjects standing with feet together, waist uncovered, arms loosely along the sides, and breathing normally [28]. The measurement was taken at the midpoint between the lowest costal bones and the highest point of the iliac crest, using a nonelastic tape with an accuracy of 0.1 cm, by the same trained health worker.

### Bioelectrical impedance analysis (BIA)

The body composition of the participants was performed by the same experienced nutritionist using a phase-sensitive BIA device (a BIA 101 RJL, Akern Bioresearch, Florence) and in standardized conditions to reduce variability between devices and between observers. In accordance with the guidelines of the European Society of Parenteral and Enteral Nutrition (ESPEN) [29], the analysis was carried out with patients on a 6-h fast without having practiced physical activity or consumed alcohol 24 h before the evaluation, as widely reported in previous research [30]. In addition, all patients were asked to empty their bladders 30 min before the BIA evaluation, as indicated by Kyle et al. [29]. The subjects were asked to remove shoes and socks for the application of the electrodes before placing themselves in a position with the limbs slightly distant from the body, and the contact surfaces were cleaned with alcohol. The electrodes (BIATRODES Akern Srl; Florence, Italy) were placed on the right hand (approximately to the phalangeal-metacarpal joint on the dorsal surface) and the right foot (distantly to the transverse arc on the upper surface of the right toe). The detection electrodes were placed on the right wrist (middle way between the distal projection of the radio and the ulna) and the right ankle (between the medial and lateral malleoli) [31]. Daily, the qualified nutritionist checked the BIA device with resistors and capacitors of known value. Under 50 kHz conditions, the phase angle (PhA) in degrees (°) was calculated using the following formula: PhA (◦, degrees) = arc tangent reactance (Xc)/resistance (R) (180/π) [32]. The analysis made it possible to evaluate the following parameters: fat mass (FM, kg and %), fat-free mass (FFM, kg and %), skeletal muscle mass (SMM, kg and %), total body water (TBW, lt and %), intracellular water (ICW, lt and %), and extra-cellular water (ECW, lt and %).

### Measurement of high sensitivity C reactive protein (hs-CRP) levels

Venous blood samples were taken in the morning, after a night's fast (at least 8 h), between 9:00 and 11:00. Hs-CRP levels were evaluated with a high-sensitivity nephelometric analysis (CardioPhase hsCRP kit, Siemens Healthcare Diagnostics, Marburg, Germany). The lower detection limit was 0.01 mg/L, and the intra- and interassay coefficients of variability was < 7%.

All subjects were further classified into three cardiovascular risk (CVR) categories based on hs-CRP levels: low CVR (< 1.0 mg/L), intermediate CVR (1.0 – 3.0 mg/L), and high CVR (≥ 3.0 mg/L) in accordance with the Centers for Disease Control and Prevention and the American Heart Association [33]**.**

### VLEKT intervention

Females and males who met the inclusion/exclusion criteria were administered a VLEKT protocol with total meal replacement, planned by the nutritionist and recommended by the endocrinologist [19]. A specialized commercial supplier (New Penta Srl, Cuneo, Italy) was used to implement VLEKT and provided high-biological-value replacement meals with whey, soy, egg, and pea proteins. The total energy intake of the diet was < 800 kcal/day and was provided by 13% carbohydrate (< 30 g/day), 43% protein (1.3 g/kg ideal body weight), and 44% fat. To maintain physiological acid/base balance, supplementation of vitamins (B complex, vitamins C and E), minerals and omega-3 fatty acids at the same dosage was provided (PentaCal, New Penta, Ltd., Cuneo, Italy) [19].

An example of a VLEKT scheme with meal replacements is report in Supplementary Materials (Supplementary Materials File S1). A skilled Nutritionist conducted individual telephone interviews with each participant in order to monitor adherence to the prescribed diet and level of physical activity. Specifically, in order to monitor the state of ketosis, the participants were asked to independently measure β-hydroxybutyrate levels by capillary blood sampling using a test strip (Optium Xceed Blood Glucose and Ketone Monitoring System; Abbott Laboratories, Chicago, IL, USA) and to report the result during the telephone interview. The same measurement of capillary β-hydroxybutyrate levels was carried out at baseline and at the end of the 45-day VLEKT period in the outpatient clinic during the nutrition visit by healthcare personnel involved in the study. Both outpatient and self-dosing by the patient were performed in the morning on an empty stomach. Similarly, during the weekly telephone interview, information was collected on adherence to the nutritional indications (intake of permitted and prohibited foods) and physical exercise (in particular, ensuring that no physical activity was practised for the duration of the nutritional programme, as indicated).

### Statistical analysis

All individuals completed 45 days of VLEKT and had performed initial, intermediate (telephone interviews) and final measurements. Data were analyzed using SPSS software and the MedCalc® package. Results were described as mean ± standard deviation (SD) or percentage (%). Data distribution was assessed using a Kolmogorov–Smirnov test. Skewed variables were normalized by a logarithm and converted back into tables and figures. Differences in variables at baseline and after VLEKT were compared using the paired Student's *t* test. The chi-square test (χ^2^) was used to determine the significance of differences in frequency distribution between BMI categories. Spearman correlation was used to evaluate the association between pre/post intervention percentage changes (delta ∆%). Odds differences are expressed as odds ratios (OR) and 95% confidence intervals (95% CI). The effects of potential confounders were removed by partial correlation. A *p*-value (two-tailed) < 0.005 was considered statistically significant.

Since this was a pilot study, no power calculations were performed. It follows that all results need to be confirmed by larger clinical studies.

## Results

Twenty-one premenopausal females and 21 males (aged 32.71 ± 8.78 and 37.76 ± 10.75 years, *p* = 0.103, respectively), who met the inclusion/exclusion criteria, were included in these statistical analyses. All study participants were evaluated at baseline and after 45 days of VLEKT. Adherence to VLEKT was assessed and confirmed in all participants by a qualified nutritionist through a telephone interview once a week. In detail, all study participants were called the day before the interview to be instructed to do the ketosis capillary test, and the results were registered on the day of the interview by the nutritionist. All participants completed the study.

Table 2 reports the anthropometric, body composition and inflammatory parameters of the study population according to gender at baseline and after 45 days of VLEKT.

**Table 2:** Baseline and post-45-day VLEKT anthropometric, body composition and inflammatory parameters of the study population according to gender

Parameters	Baseline			Post-45-day VLCKD		
	Females n. 21 (50.0%)	Males n. 21 (50.0%)	*p*	Females n. 21 (50.0%)	Males n. 21 (50.0%)	*p*
Anthropometric parameters						
Weight (kg)	92.00 ± 10.45	110.48 ± 9.86	** < 0.001**	83.78 ± 9.77	97.70 ± 9.79	** < 0.001**
BMI (kg/m^2^)	34.13 ± 2.41	35.50 ± 2.82	0.100	31.06 ± 2.08	31.39 ± 2.85	0.676
Overweight (n, %)	–	–	–	7, 33.3%	8, 38.1%	χ^2^ = 0.01, p = 0.999
Grade I obesity (n, %)	14, 66.7%	10, 47.6%	χ^2^ = 0.88, p = 0.349	13, 61.9%	11, 52.4%	χ^2^ = 0.09, p = 0.755
Grade II obesity (n, %)	7, 33.3%	11, 52.4%		1, 4.8%	2, 9.5%	χ^2^ = 0.01, p = 0.999
WC (cm)	102.19 ± 11.46	118.98 ± 11.18	** < 0.001**	93.95 ± 10.64	107 ± 10.62	** < 0.001**
< cut off *	2, 9.5%	0, 0.0%	χ^2^ = 0.53, p = 0.469	7, 33.3%	8, 38.1%	χ^2^ = 0.01, p = 0.999
> cut off *	19, 90.5%	21, 100.0%		14, 66.7%	13, 61.9%	
Body composition parameters						
R (Ω)	496.24 ± 56.77	423.43 ± 51.35	**0.008**	484.09 ± 62.97	433.00 ± 54.74	** < 0.001**
Xc (Ω)	43.95 ± 5.26	41.00 ± 7.20	0.137	45.95 ± 5.77	48.57 ± 6.50	0.137
PhA (°)	5.07 ± 0.33	5.50 ± 0.51	**0.002**	5.42 ± 0.30	6.40 ± 0.30	**0.002**
TBW (lt)	39.45 ± 4.26	55.13 ± 5.30	** < 0.001**	39.26 ± 4.50	52.33 ± 5.46	** < 0.001**
TBW (%)	43.00 ± 3.19	50.06 ± 4.48	** < 0.001**	47.01 ± 3.94	53.80 ± 5.18	** < 0.001**
ECW (lt)	19.91 ± 2.23	26.64 ± 3.59	** < 0.001**	19.03 ± 2.21	23.00 ± 2.54	** < 0.001**
ECW (%)	50.49 ± 1.92	48.20 ± 2.62	**0.002**	48.48 ± 1.51	43.94 ± 1.33	**0.002**
ICW (lt)	19.54 ± 2.27	28.50 ± 2.36	** < 0.001**	20.23 ± 2.44	29.33 ± 3.10	** < 0.001**
ICW (%)	49.51 ± 1.92	51.80 ± 2.62	**0.003**	51.51 ± 1.51	56.06 ± 1.33	**0.002**
FM (kg)	39.17 ± 7.44	37.92 ± 8.68	0.618	30.92 ± 6.68	26.60 ± 8.21	0.618
FM (%)	42.31 ± 4.29	33.99 ± 5.31	** < 0.001**	36.65 ± 4.96	26.91 ± 6.86	** < 0.001**
FFM (kg)	52.83 ± 4.92	72.57 ± 4.26	** < 0.001**	52.86 ± 5.42	71.09 ± 6.97	** < 0.001**
FFM (%)	57.68 ± 4.29	66.01 ± 5.31	** < 0.001**	36.65 ± 4.96	26.91 ± 6.86	** < 0.001**
BCM (kg)	25.70 ± 2.77	36.68 ± 2.36	** < 0.001**	26.83 ± 2.98	37.55 ± 5.22	** < 0.001**
BCMI (kg/m^2^)	9.55 ± 0.69	11.78 ± 0.70	** < 0.001**	9.96 ± 0.74	12.26 ± 1.13	** < 0.001**
SMM (kg)	24.85 ± 3.52	36.38 ± 4.78	** < 0.001**	25.50 ± 3.91	36.99 ± 6.33	** < 0.001**
SMM (%)	27.07 ± 2.76	33.08 ± 4.65	** < 0.001**	30.50 ± 3.72	38.11 ± 7.23	** < 0.001**
Inflammatory parameter						
hs-CRP levels (mg/L)	2.78 ± 0.88	2.80 ± 1.19	0.948	2.17 ± 0.81	1.62 ± 0.81	**0.034**
Low CVR^§^ (n,%)	0, 0.0%	0, 0.0%	χ^2^ = 0.01, p = 0.999	2, 9.5%	5, 23.8%	χ^2^ = 0.69, p = 0.408
Intermediate CVR^§^ (n, %)	14, 66.7%	14, 66.7%		16, 76.2%	14, 66.7%	χ^2^ = 0.12, p = 0.733
High CVR^§^ (n, %)	7, 33.3%	7, 33.3%		3, 14.3%	2, 9.5%	χ^2^ = 0.01, p = 0.999

A *p*-value in bold type denotes a significant difference (*p* < 0.05)

VLEKT, very low-energy ketogenic therapy; BMI, body mass index; WC, waist circumference; R, resistance; Xc, reactance; PhA, phase angle; TBW, total body water; ECW, extracellular water; ICW, intracellular water; FM, fat mass; FFM, fat free mass; BCM, body cell mass; BCMI, body cell mass index; SMM, skeletal muscle mass; hs-CRP, high-sensitivity C-reactive protein; CVR, cardiovascular risk

^*^WC 88 cm and 102 cm for females and males, respectively

^§^Low CVR (< 1.0 mg/L), intermediate CVR (1.0—3.0 mg/L), high CVR (≥ 3.0 mg/L)

At baseline, premenopausal females and males did not differ in BMI (*p* = 0.100), BMI categories (*p* = 0.349), hs-CRP levels (*p* = 0.948), and CVR categories (*p* = 0.999). In reverse, significant differences were evident for weight (*p* < 0.001), WC (*p* < 0.001), R (*p* = 0.008), PhA (*p* = 0.002), TBW (lt and %) (*p* < 0.001), ECW (lt) (*p* < 0.001), ECW (%) (*p* = 0.002), ICW (lt) (*p* < 0.001), ICW (%) (*p* = 0.003), FM (%) (*p* < 0.001), FFM (kg and %) (*p* < 0.001), BCM (*p* < 0.001), BCMI (*p* < 0.001), SMM (kg and %) (*p* < 0.001). Also, at post-45-day of VLEKT, the same differences in study parameters were maintained, except for hs-CRP levels, which differed between males and premenopausal females (*p* = 0.034); Table 2.

Table 3 showed the percentage changes (Δ%) in population study parameters according to gender. Premenopausal females and males significantly differed for percentage changes in BMI (*p* < 0.001), WC (*p* = 0.005), R (*p* = 0.002), Xc (*p* < 0.001), PhA (*p* < 0.001), TBW (lt) (*p* < 0.001), TBW (%) (*p* = 0.049), ECW (lt and %) (*p* < 0.001), ICW (%) (*p* < 0.001), and hs-CRP levels (*p* = 0.003).

**Table 3:** Percentage changes (Δ%) in population study parameters according to gender

Parameters	Females Subjects n. 21 (50.0%)	Males Subjects n. 21 (50.0%)	*p*
	Δ%	Δ%	
Anthropometric parameters			
Weight (kg)	− 8.95 ± 1.65	− 11.63 ± 1.76	** < 0.001**
BMI (kg/m^2^)	− 8.95 ± 1.65	− 11.63 ± 1.76	** < 0.001**
WC (cm)	− 8.06 ± 1.95	− 9.67 ± 1.50	**0.005**
Body composition parameters			
R (Ω)	− 2.49 ± 4.99	2.27 ± 4.36	**0.002**
Xc (Ω)	4.66 ± 6.01	19.56 ± 9.39	** < 0.001**
PhA (°)	7.05 ± 3.30	17.11 ± 9.00	** < 0.001**
TBW (lt)	− 0.51 ± 3.27	− 5.12 ± 2.54	** < 0.001**
TBW (%)	9.27 ± 3.36	7.43 ± 2.49	**0.049**
ECW (lt)	− 4.41 ± 3.45	− 13.32 ± 3.95	** < 0.001**
ECW (%)	− 3.94 ± 1.59	− 8.66 ± 3.87	** < 0.001**
ICW (lt)	3.54 ± 3.78	4.10 ± 1.91	0.645
ICW (%)	2.85 ± 5.66	8.41 ± 4.55	** < 0.001**
FFM (kg)	− 0.00 ± 2.73	− 2.18 ± 5.00	0.582
FFM (%)	9.84 ± 2.85	10.73 ± 5.15	0.492
BCM (kg)	4.42 ± 4.00	2.55 ± 14.13	0.564
BCMI (kg/m)	4.39 ± 3.83	4.39 ± 11.76	0.999
SMM (kg)	2.54 ± 5.22	1.44 ± 7.41	0.582
SMM (%)	12.59 ± 5.53	14.76 ± 7.77	0.303
Inflammatory parameter			
hs− CRP levels (mg/L)	− 22.38 ± 17.30	− 41.42 ± 21.35	**0.003**

A *p*-value in bold type denotes a significant difference (p < 0.05)

BMI, body mass index; WC, waist circumference; R, resistance; Xc, reactance; PhA, phase angle; TBW, total body water; ECW, extracellular water; ICW, intracellular water; FFM, fat free mass; BCM, body cell mass; BCMI, body cell mass index; SMM, skeletal muscle mass; hs-CRP, high-sensitivity C-reactive protein

**Fig. 2:**
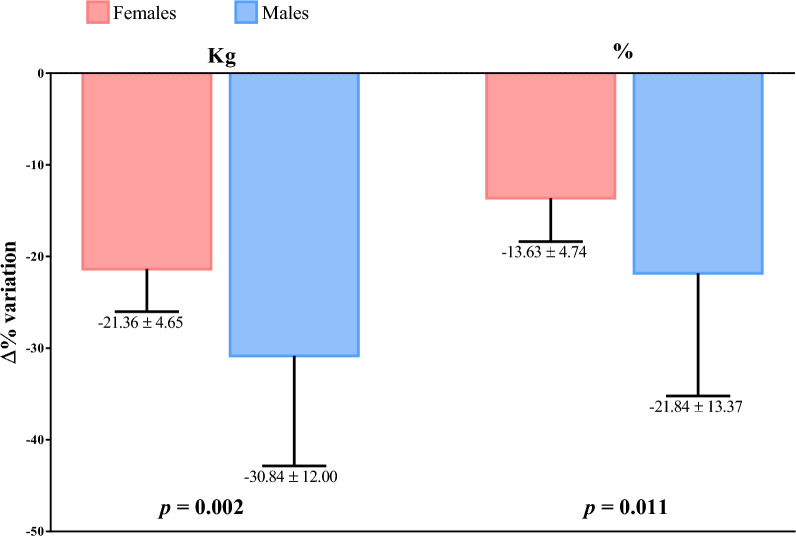
Percentage changes (Δ%) in fat mass (kg and %) according to gender

Figure 2 showed the Δ% in FM (kg and %) according to gender. Males lose more FM than premenopausal females, both in kg (*p* = 0.002) and % (*p* = 0.011).

The results of the bivariate OR model, performed to assess the association of gender with the percentage changes of study parameters, are reported in Table 4. Gender was significantly associated with the percentage changes in BMI (*p* = 0.001), WC (*p* = 0.010), R (*p* = 0.011), Xc (*p* = 0.003), PhA (*p* = 0.002), TBW (lt) (*p* = 0.001), ECW (lt and %) (*p* = 0.014 and *p* = 0.002, respectively), ICW (%) (*p* = 0.004), FM (kg and %) (*p* = 0.006 and *p* = 0.022, respectively), and hs-CRP levels (*p* = 0.006).

**Table 4:** Bivariate odds ratio (OR) model to assess the association of gender with percentage changes (Δ%) of study parameters

Parameters	OR	*p*-value	95% IC	R^2^
Δ% Age (Years)	0.95	0.105	0.88–1.01	0.064
Anthropometric parameters				
Δ% weight (kg)	2.47	**0.001**	1.46–4.19	0.382
Δ% BMI (kg/m^2^)	2.47	**0.001**	1.46–4.19	0.382
Δ% WC (cm)	1.71	**0.010**	1.14–2.57	0.180
Body composition parameters				
Δ% R (Ω)	0.42	**0.011**	0.22–0.82	0.350
Δ% Xc (Ω)	0.66	**0.003**	0.50–0.87	0.543
Δ% PhA (°)	0.66	**0.002**	0.51–0.86	0.446
Δ% TBW (lt)	0.89	**0.001**	2.42–33.48	0.628
Δ% TBW (%)	1.32	0.071	0.98–1.78	0.103
Δ% ECW (lt)	4.39	**0.014**	1.35–14.26	0.662
Δ% ECW (%)	2.38	**0.002**	1.37–4.15	0.456
Δ% ICW (lt)	1.03	0.636	0.91–1.18	0.005
Δ% ICW (%)	0.60	**0.004**	0.42–0.85	0.322
Δ% FM (kg)	1.18	**0.006**	1.05–1.33	0.258
Δ% FM (%)	1.12	**0.022**	1.02–1.24	0.173
Δ% FFM (kg)	1.15	0.096	0.98–1.37	0.072
Δ% FFM (%)	0.95	0.482	0.82–1.10	0.012
Δ% BCM (kg)	1.02	0.555	0.96–1.08	0.008
Δ% BCMI (kg/m)	1.00	0.999	0.93–1.07	0.001
Δ% SMM (kg)	1.03	0.574	0.93–1.14	0.008
Δ% SMM (%)	0.95	0.305	0.86–1.05	0.027
Inflammatory parameter				
Δ% hs-CRP levels (mg/L)	1.05	**0.006**	1.01–1.08	0.192

A *p*-value in bold type denotes a significant difference (p < 0.05)

BMI, body mass index; WC, waist circumference; R, resistance; Xc, reactance; PhA, phase angle; TBW, total body water; ECW, extracellular water; ICW, intracellular water; FM, fat mass; FFM, fat free mass; BCM, body cell mass; BCMI, body cell mass index; SMM, skeletal muscle mass; hs-CRP, high-sensitivity C-reactive protein

## Discussion

This study aimed to evaluate the effects of gender differences on weight loss, in changes in body composition and inflammatory status in patients with grade I and II obesity without metabolic comorbidities undergoing a VLEKT for 45 days. All study participants complied with the 45 days of the VLEKT protocol, obtaining significant weight loss, remodeling of body composition and reduction of the inflammatory state. Along with the expected overall improvements, we also observed that males experience overall larger benefits than premenopausal females from VLEKT in terms of weight loss, body composition improvements, and reduction of inflammation.

This was in line with a very recent study that revealed that the efficacy of adhering to a VLEKT in severe obesity is influenced by gender differences and, in the case of females, by menopausal status. In this study, males tended to experience greater benefits than pre-menopausal females in terms of excess body weight loss (EBWL) and improvement in non-alcoholic fatty liver disease (NAFLD). However, these differences become less pronounced after menopause, likely due to changes in hormonal profiles and body composition. In contrast, post-menopausal females emerged as the group that derived the least benefit from following the VLEKT. However, limitations of the study, including the small sample size, particularly the underrepresented male group, affected the generalizability results [23]. Notably, our study extended this understanding by demonstrating that improvements in body composition along with reductions in inflammation measured by hs-CRP, were more pronounced in males compared to females following the VLEKT protocol. Additionally, we provided insights into how factors such as baseline body composition, hormonal profiles, and hydration status may influence these gender-specific responses to dietary interventions in obesity management. These findings highlight the importance of considering sex differences in personalized approaches to obesity treatment, particularly regarding dietary interventions like VLEKTs.

At baseline, in our study population, females exhibited lower weight, WC, and SMM (kg and %) and higher FM (%) and FFM (kg and %) compared to males. A higher percentage of males fell into the obesity class II category in contrast to females. The prevalence of obesity varies globally, with socio-economic factors influencing patterns [7]. In the U.S. (NHANES 2005–2014), women had a higher obesity rate (40%) than men (35%), while Italy (2016) showed a reversal, with men having a higher rate (51% *vs.* 34% in women) [34, 35]. In addition, it is already known that women have more body fat and less muscle mass than men. Compared with men, women also have more subcutaneous fat, particularly in the abdominal and gluteal-femoral areas, and less visceral fat [36].

In our study population, females also showed higher R and lower PhA compared to males at baseline. In these regards, evidence reports that gender primarily influences variability in R, with higher values in females than in males at all frequencies [37–39]. PhA is the ratio of Xc to R and describes the relationship between ICW and ECW, or between BCM and extracellular mass. PhA is one of the best indicators of cellular health. Studies suggest that the higher the PhA, the stronger the cell membrane and the better the cell function [40, 41]. Generally, PhA increases with good health, proper nutrition, and exercise, but also in males [42]. Higher PhA in males than in females is probably due to greater BCM and thus greater SMM (the largest intracellular water reservoir) often seen in males [43], as also shown in our results.

As for hydration and body water spaces, females showed lower TBW (lt and %), ECW (lt) and ICW (lt and %) but higher ECW (%) compared to males. Obesity is characterized by alterations in hydration and body water spaces. It was previously demonstrated that as people increase in BMI from normality to obesity, TBW makes a significantly lower proportion of weight [44]. In addition, there is a gender difference in this parameter, and at the same BMI, females have less water *per* kg of body weight than males [44]. In obesity, apart from an increase in FM and, to a lesser extent, increased FFM, the ECW compartment is enlarged compared with the ICW compartment. Proposed explanations for the high ECW/ICW ratio are a high ECW/ICW of the adipose tissue [45, 46], obesity-related edema, and hormonal responses related to the adipose tissue [47]. Thus, looking at the previously presented percentage showing higher FM in females could also explain the higher ECW content found in females compared to males.

Examining the percentage changes (Δ%) in our population study parameters by gender, females compared to males exhibited the following:

- Significantly lower decreases in BMI, WC, and FM (kg and %).

We can attempt to elucidate these discrepancies considering the hypothesized mechanisms of action of VLEKT, although this remains largely unknown. Fundamentally, VLEKTs aim to induce a metabolic shift from carbohydrates to triglycerides stored in adipose tissue, establishing them as the primary energy source for basal metabolism [48]. Notably, visceral adipose tissue exhibits greater metabolic activity compared to subcutaneous visceral fat, characterized by heightened lipolysis and increased release of free fatty acids [49]. Considering that males have more visceral fat, as also evidenced in our population with higher baseline WC than females, and higher basal energy expenditure due to higher lean mass, as found in our results with higher baseline FFM and SMM in male participants, these factors could contribute to the more significant benefits observed in males adhering to a VLEKT, especially in terms of body weight loss and improved body composition.

Studies have also found some evidence that specific dietary approaches may have differential effects by gender [46, 47, 50, 51]. For example, one study of adults randomized to either healthy low-carbohydrate (HLC) or healthy low-fat (HLF) for 12 months found that males were more likely to adhere to HLC diets than females and that females had higher adherence to HLF diets than HLC diets [50]. Men had more success with HLC diets than HLF diets, while women did not demonstrate a significant difference in success between the two diets [50]. Multiple studies also assessed gender differences following low-energy diets or very-low energy diets, with many findings that males were more successful in achieving higher percent weight loss [51–53].

b) Percentage changes in R varied significantly between females and males, decreasing in the former and increasing in the latter. For Xc and PhA, the increases were also significantly lower in females than in males.
  Our previous results emphasized that changes in PhA during the active phase of VLEKT occurred early in treatment and were not influenced by confounding variables, particularly weight loss [16, 54]. Based on these results, we unveil an interesting aspect: gender significantly influences PhA improvement during VLEKT, showing more pronounced improvement in males, even when they start with higher baseline values than females.

c) Significantly lower decreases in TBW (lt) and ECW (lt and %), significantly lower increases in ICW (%) and significantly higher increases in TBW (%),

d) Significantly lower decreases in hs-CRP levels.

Water is vital for life, the major constituent of the human body, and the main element of cells, tissues, and organs [55]. Furthermore, water is an excellent solvent, reaction medium, reactant, and reaction product; it is a carrier of nutrients and waste products, involved in thermoregulation, and a lubricant and shock absorber [56]. Thus, fluid balance is critical in health and disease and involved in stress- and inflammation-induced changes during the development of various acute or chronic diseases [57–60]. Of note, ECW has attracted interest as an inflammatory marker. In a large multicenter retrospective study of 9246 subjects, ECW has been associated with medically unexplained symptoms a persistent psychosomatic bodily complaint, employed as a clinical index of chronic stress and inflammation [61]. In peritoneal dialysis patients, inflammation has been related to extracellular fluid volume expansion [62–64]. It has been proposed that expanded extracellular volume, due to inadequate water and sodium removal, acts as an inflammatory stimulus in these patients [65, 66]. On the other hand, the inflammatory process itself may promote ECW expansion, as seen in patients with peritonitis. In addition, in the general population, CRP, is also considered to be a marker of inflammation, and a powerful risk factor for ischemic heart disease and peripheral atherosclerosis [67]. In our investigation, we observed a more pronounced reduction in inflammatory status, measured by hs-CRP, among males compared to females. While the greater reduction in WC and FM and the substantial improvement in PhA in males contribute to this phenomenon, we propose that the noteworthy decrease in ECW in males at the conclusion of the VLEKT could be a pivotal factor.

### Limitations and strengths

Our study has several strengths but also limitations. First, this pilot study of an adult population involved a single-center analysis that may have introduced some selection bias. However, we adopted strict inclusion criteria for selecting study participants. These criteria aimed to create a homogeneous and representative sample to avoid the influence of external factors that could alter the results. Second, as a pilot study, we did not extend our observations to the subsequent VLEKT stages. However, due to the very short observation period, it was possible to avoid potential patient dropouts. Thirdly, there was no statistical analysis of the side effects of VLEKT, although all safety parameters were monitored during the endocrinological and nutritional assessments. However, the lack of this analysis cannot have influenced the results of the study, since, as we have previously shown in a study on a larger number of subjects [68], when appropriately designed and prescribed, VLEKT represents a safe dietary intervention that does not lead to major alterations in haematochemical parameters, and side effects are minimal, mostly transient, and do not occur according to a gender-specific trend. Furthermore, hormone levels that could potentially influence weight loss were not measured, limiting the assessment of their impact on study outcomes. For strengths, BIA is a widely used and valid method for the assessment of body composition, including obesity. It is noninvasive, rapid, and relatively simple to perform, making it a practical choice in clinical and research settings. As a direct BIA measurement, PhA is not influenced by altered tissue hydration in the algorithmic computations to assess other body composition compartments [54]. Another strength of this study was the performance and interpretation of BIA measurements by only one clinical nutritionist to minimize inter-operator variability. Hs-CRP is widely recognized as the most used marker of inflammation [69], adding robustness to the study's assessment of inflammatory status. Finally, adherence to VLEKT was evaluated weekly, ensuring the maintenance of ketosis and consistency in lifestyle throughout the study period.

## Conclusion

These findings underscore the importance of accounting for gender-based variations when assessing the effects of VLEKT in individuals with obesity. This recognition is critical for developing personalized strategies to address obesity-related conditions. It also underscores the need to take into account the different inflammatory states of individuals with obesity. By confirming these results with studies on a larger number of subjects of different age groups, thus, useful information could be obtained that would make it possible to identify, differentiating between males and females, both the optimal weight reduction target and the expected changes in weight, body composition and inflammation following a VLEKT protocol. As a consequence, this would make it possible to optimize the nutritional intervention in terms of composition and/or duration in order to achieve the gender-specific nutritional goals. By recognizing and incorporating these factors, we can improve our understanding of the impact of VLEKT and refine interventions to better fit individual needs and conditions.

## Supplementary Information

Supplementary material 1.

## Data Availability

The datasets used and/or analysed during the current study are available from the corresponding author on reasonable request.

## References

[1] Organization WH. The programme for gender equality, human rights & health equity. 2024; https://www.who.int/teams/gender-equity-and-human-rights.

[2] Mauvais-Jarvis F, Bairey Merz N, Barnes PJ, et al. Sex and gender: modifiers of health, disease, and medicine. Lancet. 2020;396(10250):565–82. 10.1016/S0140-6736(20)31561-0. 32828189 10.1016/S0140-6736(20)31561-0 PMC7440877

[3] Cooper AJ, Gupta SR, Moustafa AF, Chao AM. Sex/gender differences in obesity prevalence, comorbidities, and treatment. Curr Obes Rep. 2021;10(4):458–66. 10.1007/s13679-021-00453-x. 34599745 10.1007/s13679-021-00453-x

[4] Barrea L, Verde L, Suarez R, et al. Sex-differences in Mediterranean diet: a key piece to explain sex-related cardiovascular risk in obesity? A cross-sectional study. J Transl Med. 2024;22(1):44. 10.1186/s12967-023-04814-z. 38200498 10.1186/s12967-023-04814-z PMC10782790

[5] Organization WH. Obesity and overweight. 2023; https://www.who.int/news-room/fact-sheets/detail/obesity-and-overweight.

[6] Martin M, Beekley A, Kjorstad R, Sebesta J. Socioeconomic disparities in eligibility and access to bariatric surgery: a national population-based analysis. Surg Obes Relat Dis. 2010;6(1):8–15. 10.1016/j.soard.2009.07.003. 19782647 10.1016/j.soard.2009.07.003

[7] Muscogiuri G, Verde L, Vetrani C, Barrea L, Savastano S, Colao A. Obesity: a gender-view. J Endocrinol Invest. 2023. 10.1007/s40618-023-02196-z. 37740888 10.1007/s40618-023-02196-z PMC10859324

[8] Gerdts E, Regitz-Zagrosek V. Sex differences in cardiometabolic disorders. Nat Med. 2019;25(11):1657–66. 10.1038/s41591-019-0643-8. 31700185 10.1038/s41591-019-0643-8

[9] Imhof A, Frohlich M, Loewel H, et al. Distributions of C-reactive protein measured by high-sensitivity assays in apparently healthy men and women from different populations in Europe. Clin Chem. 2003;49(4):669–72. 10.1373/49.4.669. 12651827 10.1373/49.4.669

[10] Khera A, McGuire DK, Murphy SA, et al. Race and gender differences in C-reactive protein levels. J Am Coll Cardiol. 2005;46(3):464–9. 10.1016/j.jacc.2005.04.051. 16053959 10.1016/j.jacc.2005.04.051

[11] Lakoski SG, Cushman M, Criqui M, et al. Gender and C-reactive protein: data from the multiethnic study of atherosclerosis (MESA) cohort. Am Heart J. 2006;152(3):593–8. 10.1016/j.ahj.2006.02.015. 16923436 10.1016/j.ahj.2006.02.015

[12] Williams RL, Wood LG, Collins CE, Callister R. Effectiveness of weight loss interventions–is there a difference between men and women: a systematic review. Obes Rev. 2015;16(2):171–86. 10.1111/obr.12241. 25494712 10.1111/obr.12241 PMC4359685

[13] Robertson C, Avenell A, Boachie C, et al. Should weight loss and maintenance programmes be designed differently for men? A systematic review of long-term randomised controlled trials presenting data for men and women: the ROMEO project. Obes Res Clin Pract. 2016;10(1):70–84. 10.1016/j.orcp.2015.04.005. 25937165 10.1016/j.orcp.2015.04.005

[14] Barrea L, Verde L, Camajani E, et al. Effects of very low-calorie ketogenic diet on hypothalamic-pituitary-adrenal axis and renin-angiotensin-aldosterone system. J Endocrinol Invest. 2023;46(8):1509–20. 10.1007/s40618-023-02068-6. 37017918 10.1007/s40618-023-02068-6 PMC10349006

[15] Barrea L, Verde L, Di Lorenzo C, Savastano S, Colao A, Muscogiuri G. Can the ketogenic diet improve our dreams? Effect of very low-calorie ketogenic diet (VLCKD) on sleep quality. J Transl Med. 2023;21(1):479. 10.1186/s12967-023-04280-7. 37464397 10.1186/s12967-023-04280-7 PMC10353204

[16] Barrea L, Verde L, Santangeli P, et al. Very low-calorie ketogenic diet (VLCKD): an antihypertensive nutritional approach. J Transl Med. 2023;21(1):128. 10.1186/s12967-023-03956-4. 36800966 10.1186/s12967-023-03956-4 PMC9936635

[17] Camajani E, Feraco A, Verde L, et al. Ketogenic diet as a possible non-pharmacological therapy in main endocrine diseases of the female reproductive system: a practical guide for nutritionists. Curr Obes Rep. 2023;12(3):231–49. 10.1007/s13679-023-00516-1. 37405618 10.1007/s13679-023-00516-1 PMC10482777

[18] Camajani E, Feraco A, Proietti S, et al. Very low calorie ketogenic diet combined with physical interval training for preserving muscle mass during weight loss in sarcopenic obesity: a pilot study. Front Nutr. 2022;9: 955024. 10.3389/fnut.2022.955024. 36245515 10.3389/fnut.2022.955024 PMC9560671

[19] Muscogiuri G, El Ghoch M, Colao A, et al. European guidelines for obesity management in adults with a very low-calorie ketogenic diet: a systematic review and meta-analysis. Obes Facts. 2021;14(2):222–45. 10.1159/000515381. 33882506 10.1159/000515381 PMC8138199

[20] Barrea L, Caprio M, Camajani E, et al. Ketogenic nutritional therapy (KeNuT)-a multi-step dietary model with meal replacements for the management of obesity and its related metabolic disorders: a consensus statement from the working group of the Club of the Italian Society of Endocrinology (SIE)-diet therapies in endocrinology and metabolism. J Endocrinol Invest. 2024. 10.1007/s40618-023-02258-2. 38238506 10.1007/s40618-023-02258-2 PMC10904420

[21] Barrea L, Verde L, Schiavo L, et al. Very Low-Calorie Ketogenic Diet (VLCKD) as Pre-Operative First-Line Dietary Therapy in Patients with Obesity Who Are Candidates for Bariatric Surgery. *Nutrients.* 2023;15(8). 10.3390/nu15081907. 10.3390/nu15081907 PMC10142118 37111126

[22] Barrea L, Caprio M, Grassi D, et al. A new nomenclature for the very low-calorie ketogenic diet (VLCKD): very low-energy ketogenic therapy (VLEKT). Ketodiets and nutraceuticals expert panels: “KetoNut”, Italian Society of Nutraceuticals (SINut) and the Italian Association of Dietetics and Clinical Nutrition (ADI). Curr Nutr Rep. 2024;13(3):552–6. 10.1007/s13668-024-00560-w. 39039372 10.1007/s13668-024-00560-w PMC11327192

[23] D’Abbondanza M, Ministrini S, Pucci G, et al. Very low-carbohydrate ketogenic diet for the treatment of severe obesity and associated non-alcoholic fatty liver disease: the role of sex differences. Nutrients. 2020. 10.3390/nu12092748. 32916989 10.3390/nu12092748 PMC7551320

[24] Rinaldi R, De Nucci S, Donghia R, et al. Gender differences in liver steatosis and fibrosis in overweight and obese patients with metabolic dysfunction-associated steatotic liver disease before and after 8 weeks of very low-calorie ketogenic diet. Nutrients. 2024. 10.3390/nu16101408. 38794646 10.3390/nu16101408 PMC11123918

[25] Barrea L, Verde L, Simancas-Racines D, et al. Adherence to the Mediterranean diet as a possible additional tool to be used for screening the metabolically unhealthy obesity (MUO) phenotype. J Transl Med. 2023;21(1):675. 10.1186/s12967-023-04546-0. 37770999 10.1186/s12967-023-04546-0 PMC10540328

[26] Barrea L, Verde L, Vetrani C, Savastano S, Colao A, Muscogiuri G. Chronotype: A Tool to Screen Eating Habits in Polycystic Ovary Syndrome? Nutrients. 2022. 10.3390/nu14050955. 35267930 10.3390/nu14050955 PMC8912410

[27] Obesity: preventing and managing the global epidemic. Report of a WHO consultation. World Health Organ Tech Rep Ser*.* 2000;894:i-xii, 1–253. 11234459

[28] Prevention CfDCa. National health and nutrition examination survey: anthropometry procedures manual. 2007; https://www.cdc.gov/nchs/data/nhanes/nhanes_07_08/manual_an.pdf.

[29] Kyle UG, Bosaeus I, De Lorenzo AD, et al. Bioelectrical impedance analysis-part II: utilization in clinical practice. Clin Nutr. 2004;23(6):1430–53. 10.1016/j.clnu.2004.09.012. 15556267 10.1016/j.clnu.2004.09.012

[30] Barrea L, Muscogiuri G, Laudisio D, et al. Phase angle: a possible biomarker to quantify inflammation in subjects with Obesity and 25(OH)D deficiency. Nutrients. 2019. 10.3390/nu11081747. 31362440 10.3390/nu11081747 PMC6723101

[31] Bera TK. Bioelectrical impedance methods for noninvasive health monitoring: a review. J Med Eng. 2014;2014: 381251. 10.1155/2014/381251. 27006932 10.1155/2014/381251 PMC4782691

[32] Kumar S, Dutt A, Hemraj S, Bhat S, Manipadybhima B. Phase angle measurement in healthy human subjects through bio-impedance analysis. Iran J Basic Med Sci. 2012;15(6):1180–4. 23653848 PMC3646229

[33] Pearson TA, Mensah GA, Alexander RW, et al. Markers of inflammation and cardiovascular disease: application to clinical and public health practice: a statement for healthcare professionals from the centers for disease control and prevention and the American Heart Association. Circulation. 2003;107(3):499–511. 10.1161/01.cir.0000052939.59093.45. 12551878 10.1161/01.cir.0000052939.59093.45

[34] Flegal KM, Kruszon-Moran D, Carroll MD, Fryar CD, Ogden CL. Trends in obesity among adults in the United States, 2005 to 2014. JAMA. 2016;315(21):2284–91. 10.1001/jama.2016.6458. 27272580 10.1001/jama.2016.6458 PMC11197437

[35] 2015 IOoHR. Health status and quality of care in the Italian regions. In:2015.

[36] Chang E, Varghese M, Singer K. Gender and sex differences in adipose tissue. Curr Diab Rep. 2018;18(9):69. 10.1007/s11892-018-1031-3. 30058013 10.1007/s11892-018-1031-3 PMC6525964

[37] Biasioli S, Foroni R, Petrosino L, et al. Effect of aging on the body composition of dialyzed subjects. Comp Normal Sub ASAIO J. 1993;39(3):M596-601. 8268607

[38] Chumlea WC, Guo SS, Cockram DB, Siervogel RM. Mechanical and physiologic modifiers and bioelectrical impedance spectrum determinants of body composition. Am J Clin Nutr. 1996;64(3 Suppl):413S-422S. 10.1093/ajcn/64.3.413S. 8780357 10.1093/ajcn/64.3.413S

[39] Denti L, Pasolini G, Sanfelici L, et al. Effects of aging on dehydroepiandrosterone sulfate in relation to fasting insulin levels and body composition assessed by bioimpedance analysis. Metabolism. 1997;46(7):826–32. 10.1016/s0026-0495(97)90130-x. 9225839 10.1016/s0026-0495(97)90130-x

[40] Mattar JA. Application of total body bioimpedance to the critically ill patient. Brazilian Group for Bioimpedance Study. New Horiz. 1996;4(4):493–503. 8968982

[41] Zdolsek HJ, Lindahl OA, Sjoberg F. Non-invasive assessment of fluid volume status in the interstitium after haemodialysis. Physiol Meas. 2000;21(2):211–20. 10.1088/0967-3334/21/2/301. 10847188 10.1088/0967-3334/21/2/301

[42] Stobaus N, Pirlich M, Valentini L, Schulzke JD, Norman K. Determinants of bioelectrical phase angle in disease. Br J Nutr. 2012;107(8):1217–20. 10.1017/S0007114511004028. 22309898 10.1017/S0007114511004028

[43] Dittmar M. Reliability and variability of bioimpedance measures in normal adults: effects of age, gender, and body mass. Am J Phys Anthropol. 2003;122(4):361–70. 10.1002/ajpa.10301. 14614757 10.1002/ajpa.10301

[44] Ritz P, Vol S, Berrut G, Tack I, Arnaud MJ, Tichet J. Influence of gender and body composition on hydration and body water spaces. Clin Nutr. 2008;27(5):740–6. 10.1016/j.clnu.2008.07.010. 18774628 10.1016/j.clnu.2008.07.010

[45] DiGirolamo M, Owens JL. Water content of rat adipose tissue and isolated adipocytes in relation to cell size. Am J Physiol. 1976;231(5 Pt. 1):1568–72. 10.1152/ajplegacy.1976.231.5.1568. 998803 10.1152/ajplegacy.1976.231.5.1568

[46] Wang J, Pierson RN Jr. Disparate hydration of adipose and lean tissue require a new model for body water distribution in man. J Nutr. 1976;106(12):1687–93. 10.1093/jn/106.12.1687. 993849 10.1093/jn/106.12.1687

[47] Waki M, Kral JG, Mazariegos M, Wang J, Pierson RN Jr, Heymsfield SB. Relative expansion of extracellular fluid in obese vs. nonobese women. Am J Physiol. 1991;261(2 Pt 1):199–203. 10.1152/ajpendo.1991.261.2.E199. 10.1152/ajpendo.1991.261.2.E199 1872382

[48] Paoli A, Rubini A, Volek JS, Grimaldi KA. Beyond weight loss: a review of the therapeutic uses of very-low-carbohydrate (ketogenic) diets. Eur J Clin Nutr. 2013;67(8):789–96. 10.1038/ejcn.2013.116. 23801097 10.1038/ejcn.2013.116 PMC3826507

[49] Ibrahim MM. Subcutaneous and visceral adipose tissue: structural and functional differences. Obes Rev. 2010;11(1):11–8. 10.1111/j.1467-789X.2009.00623.x. 19656312 10.1111/j.1467-789X.2009.00623.x

[50] Aronica L, Rigdon J, Offringa LC, Stefanick ML, Gardner CD. Examining differences between overweight women and men in 12-month weight loss study comparing healthy low-carbohydrate vs. low-fat diets. Int J Obes. 2021;45(1):225–34. 10.1038/s41366-020-00708-y. 10.1038/s41366-020-00708-y PMC7752762 33188301

[51] Christensen P, Meinert Larsen T, Westerterp-Plantenga M, et al. Men and women respond differently to rapid weight loss: Metabolic outcomes of a multi-centre intervention study after a low-energy diet in 2500 overweight, individuals with pre-diabetes (PREVIEW). Diabetes Obes Metab. 2018;20(12):2840–51. 10.1111/dom.13466. 30088336 10.1111/dom.13466 PMC6282840

[52] Sumithran P, Purcell K, Kuyruk S, Proietto J, Prendergast LA. Combining biological and psychosocial baseline variables did not improve prediction of outcome of a very-low-energy diet in a clinic referral population. Clin Obes. 2018;8(1):30–8. 10.1111/cob.12229. 29119687 10.1111/cob.12229

[53] Guo X, Xu Y, He H, et al. Effects of a meal replacement on body composition and metabolic parameters among subjects with overweight or obesity. J Obes. 2018;2018:2837367. 10.1155/2018/2837367. 30687550 10.1155/2018/2837367 PMC6327254

[54] Barrea L, Muscogiuri G, Aprano S, et al. Phase angle as an easy diagnostic tool for the nutritionist in the evaluation of inflammatory changes during the active stage of a very low-calorie ketogenic diet. Int J Obes (Lond). 2022;46(9):1591–7. 10.1038/s41366-022-01152-w. 35614205 10.1038/s41366-022-01152-w PMC9130054

[55] Lang F, Busch GL, Ritter M, et al. Functional significance of cell volume regulatory mechanisms. Physiol Rev. 1998;78(1):247–306. 10.1152/physrev.1998.78.1.247. 9457175 10.1152/physrev.1998.78.1.247

[56] Chambers JM. Graphical methods for data analysis*.* 1983.

[57] Jequier E, Constant F. Water as an essential nutrient: the physiological basis of hydration. Eur J Clin Nutr. 2010;64(2):115–23. 10.1038/ejcn.2009.111. 19724292 10.1038/ejcn.2009.111

[58] Popkin BM, D’Anci KE, Rosenberg IH. Water, hydration, and health. Nutr Rev. 2010;68(8):439–58. 10.1111/j.1753-4887.2010.00304.x. 20646222 10.1111/j.1753-4887.2010.00304.x PMC2908954

[59] Wong LL, Verbalis JG. Systemic diseases associated with disorders of water homeostasis. Endocrinol Metab Clin North Am. 2002;31(1):121–40. 10.1016/s0889-8529(01)00007-x. 12055984 10.1016/s0889-8529(01)00007-x

[60] Adler SM, Verbalis JG. Disorders of body water homeostasis in critical illness. Endocrinol Metab Clin North Am. 2006;35(4):873–94. 10.1016/j.ecl.2006.09.011. 17127152 10.1016/j.ecl.2006.09.011

[61] Chrousos GP, Papadopoulou-Marketou N, Bacopoulou F, Lucafo M, Gallotta A, Boschiero D. Photoplethysmography (PPG)-determined heart rate variability (HRV) and extracellular water (ECW) in the evaluation of chronic stress and inflammation. Hormones. 2022;21(3):383–90. 10.1007/s42000-021-00341-y. 35028916 10.1007/s42000-021-00341-y

[62] Vicente-Martinez M, Martinez-Ramirez L, Munoz R, et al. Inflammation in patients on peritoneal dialysis is associated with increased extracellular fluid volume. Arch Med Res. 2004;35(3):220–4. 10.1016/j.arcmed.2004.01.003. 15163463 10.1016/j.arcmed.2004.01.003

[63] Woodrow G, Oldroyd B, Wright A, et al. Abnormalities of body composition in peritoneal dialysis patients. Perit Dial Int. 2004;24(2):169–75. 15119638

[64] Plum J, Schoenicke G, Kleophas W, et al. Comparison of body fluid distribution between chronic haemodialysis and peritoneal dialysis patients as assessed by biophysical and biochemical methods. Nephrol Dial Transplant. 2001;16(12):2378–85. 10.1093/ndt/16.12.2378. 11733630 10.1093/ndt/16.12.2378

[65] Asghar RB, Green S, Engel B, Davies SJ. Relationship of demographic, dietary, and clinical factors to the hydration status of patients on peritoneal dialysis. Perit Dial Int. 2004;24(3):231–9. 15185771

[66] Avila-Diaz M, Ventura MD, Valle D, et al. Inflammation and extracellular volume expansion are related to sodium and water removal in patients on peritoneal dialysis. Perit Dial Int. 2006;26(5):574–80. 16973513

[67] Mendall MA, Patel P, Ballam L, Strachan D, Northfield TC. C reactive protein and its relation to cardiovascular risk factors: a population based cross sectional study. BMJ. 1996;312(7038):1061–5. 10.1136/bmj.312.7038.1061. 8616412 10.1136/bmj.312.7038.1061 PMC2350910

[68] Barrea L, Verde L, Vetrani C, et al. VLCKD: a real time safety study in obesity. J Transl Med. 2022;20(1):23. 10.1186/s12967-021-03221-6. 34998415 10.1186/s12967-021-03221-6 PMC8742928

[69] Jialal I, Devaraj S. Inflammation and atherosclerosis: the value of the high-sensitivity C-reactive protein assay as a risk marker. Am J Clin Pathol. 2001;116(Suppl):S108-115. 10.1309/J63V-5LTH-WYFC-VDR5. 11993695 10.1309/J63V-5LTH-WYFC-VDR5

